# Cell Growth and Division Shape mRNA–Protein Correlations

**DOI:** 10.64898/2026.05.04.722628

**Published:** 2026-05-06

**Authors:** Kuheli Biswas, Michael Sheinman, Leonardo A Sepúlveda, Ido Golding, Ariel Amir

**Affiliations:** 1 Department of Physics of Complex Systems, Weizmann Institute of Science, Rehovot, Israel; 2 Howard Hughes Medical Institute, Harvard University, Cambridge, Massachusetts, USA; 3 Department of Chemistry and Chemical Biology, Harvard University, Cambridge, Massachusetts, USA; 4 Department of Physics, Harvard University, Cambridge, Massachusetts, USA; 5 Department of Physics, University of Illinois at Urbana-Champaign, Urbana, Illinois, USA

## Abstract

Correlations between cellular variables, such as gene-expression levels, provide insights into regulatory mechanisms. We focus here on correlations between mRNA and protein levels and re-examine previously derived analytical predictions. We test this prediction on single-cell *E. coli* data and see substantial disagreement. We hypothesize that this discrepancy arises from the assumption of constant cell volume and develop a theoretical framework for mRNA–protein correlations in growing and dividing cells. Within this framework, we derive an analytical expression for mRNA–protein correlations and show that explicit incorporation of growth and division substantially alters these correlations. The resulting relation is invariant to upstream transcriptional dynamics, and we validate it using stochastic simulations across multiple gene-regulatory architectures. Finally, we show that the derived predictions are consistent with the *E. coli* data.

## 2 Introduction

Gene expression is a fundamental process in cellular physiology, governed by a complex regulatory network of mRNAs and proteins. The resulting network dynamics generate rich correlation structures among mRNA and protein levels, which can reveal features of the underlying gene-regulatory mechanisms. These correlations are further modulated by noise, which may exert opposing effects. In the classical dual-reporter setup of Elowitz *et al*. [1], intrinsic noise decreases the correlation between reporter expression levels. In other network architectures, however, intrinsic noise of an upstream regulator can propagate through a genetic cascade, thereby increasing correlations between downstream expression levels [2, 3, 4]. In these and other related studies [5, 6, 7], specific, relatively small regulatory networks were analyzed, leaving unresolved the structure of correlations and their relationship to intrinsic noise in generic regulatory circuits with many interacting components, feedback loops, and stochastic molecular processes.

To this end, seminal studies have derived exact relations between measurable quantities that depend only on a limited subset of interactions [8, 9, 10]. It was shown that mRNA–protein correlations obey a universal relation that is independent of kinetic schemes of mRNA production and are determined solely by protein production and degradation dynamics [8]. Specifically, when the translation rate is proportional to the mRNA abundance, the correlation between mRNA copy number, M, and protein copy number, P, is predicted by [8]

(1) $$\rho_{MP}=\frac{{\text{CV}}_{P}}{{\text{CV}}_{M}}\left(1-\frac{1}{{\text{CV}}_{P}^{2}\langle P\rangle }\right)\equiv {ℛ}_{0},$$

which depends on the mean protein copy number ⟨P⟩, and the intrinsic noise in protein and mRNA copy numbers, respectively, denoted by ${\text{CV}}_{P}$ and ${\text{CV}}_{M}$.

In this study, we reexamine Eq. (1) and show that it is inconsistent with single-cell *E. coli* data [11]. We hypothesized that this discrepancy arises from the assumption of constant-volume, made in Ref. [8]. This interpretation is supported by earlier studies showing that cell growth and division can substantially alter the statistics of mRNA and protein abundance relative to constant-volume models [12, 13, 14, 15, 16, 17]. Here, we develop a general framework for deriving mRNA–protein correlations for a gene embedded in an arbitrary regulatory network while explicitly accounting for cell growth and division. Within this framework, we find in simulations that Eq. (1) does not hold, and that this discrepancy persists even when the relation is reformulated in terms of concentrations. We then derive an expression for the mRNA–protein correlation in growing and dividing cells and validate this prediction using stochastic simulations across several upstream transcriptional network architectures. Finally, we test our prediction against the same single-cell *E. coli* data [11] and find good agreement with the measurements.

## 3 Results

### 3.1 Comparison of the constant-volume model with experimental data

**Figure 1:**
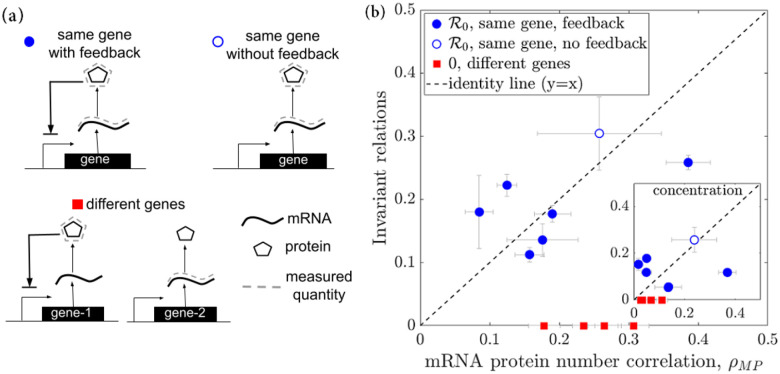
Measured mRNA–protein correlations in *E. coli* deviate from the constant-volume prediction of Eq. (1). **(a)** Schematic of the three classes of mRNA–protein pairs analyzed in the *E. coli* dataset [11]: same-gene pairs with feedback (blue filled circles), same-gene pairs without feedback (blue open circles), and different-gene pairs (red filled squares). **(b)** Comparison between measured mRNA–protein copy-number correlations, $\rho_{MP}$, and the constant-volume prediction ${ℛ}_{0}$ in Eq. (1) for same-gene pairs (blue circles). Error bars indicate bootstrap standard deviations obtained by resampling individual cells. The dashed line denotes y=x, corresponding to perfect agreement between prediction and measurement. For different-gene pairs, the constant-volume model predicts zero correlation, provided that the two genes neither regulate one another nor share a common regulator. In contrast, the experimental data exhibit substantial nonzero mRNA–protein correlations for these pairs (red squares). In the inset, we plot the same, but for mRNA and protein concentrations instead of copy numbers.

We begin by comparing Eq. (1), derived from the constant-volume model, with experimental data. To this end, we use single-cell measurements of mRNA and protein copy numbers in *E. coli* across multiple growth conditions and regulatory architectures, previously reported in Ref. [11]. The dataset contains two types of mRNA–protein pairs: *(i)* pairs in which the mRNA and protein were measured for the same gene (hereafter referred to as same-gene pairs), and *(ii)* pairs in which the mRNA and protein were measured for different genes (hereafter referred to as different-gene pairs). For same-gene pairs, measurements are available both in the presence and absence of protein-mediated feedback regulation. The corresponding regulatory architectures are shown in Fig. 1(a). In the experiment [11], protein copy numbers were quantified using immunofluorescence, whereas mRNA copy numbers were measured by single-molecule fluorescence *in situ* hybridization.

The constant-volume model developed in Ref. [8] predicts that same-gene mRNA–protein pairs should satisfy Eq. (1), whereas different-gene pairs should exhibit zero correlation, provided that the two genes neither regulate one another nor share a common regulator. However, when tested against the experimental data, for same-gene pairs, Eq. (1) fails in 2 of the 7 growth conditions examined, with Bonferroni-corrected p-values of 0.03 and 0.01. Moreover, for different-gene pairs, the constant-volume prediction of zero correlation clearly fails in all four studied growth conditions (all p-values are below 10^−7^). A summary of these results is shown in Fig. 1(b). One might argue heuristically that Eq. (1) could be adapted by replacing mRNA and protein abundances with their concentrations. However, as shown in the inset of Fig. 1(b), this concentration-based relation is also inconsistent with the data, both for same-gene pairs (4 growth conditions with Bonferroni-corrected $p<10^{-3}$) and for different-gene pairs (2 growth conditions with $p<10^{-3}$).

Several factors may contribute to these discrepancies, including limited sample size and possible experimental artifacts. Nevertheless, we hypothesize that the dominant source of the disagreement is the assumption of constant cell volume under which Eq. (1) was derived [8]. The remainder of this article is devoted to establishing this hypothesis and analyzing its consequences. In the following section, we derive the mRNA–protein copy-number correlation for a gene embedded in a regulatory network while explicitly accounting for cell growth and division, and compare the resulting prediction with simulations and the experimental data.

### 3.2 Invariant relations for growing and dividing cells

**Figure 2:**
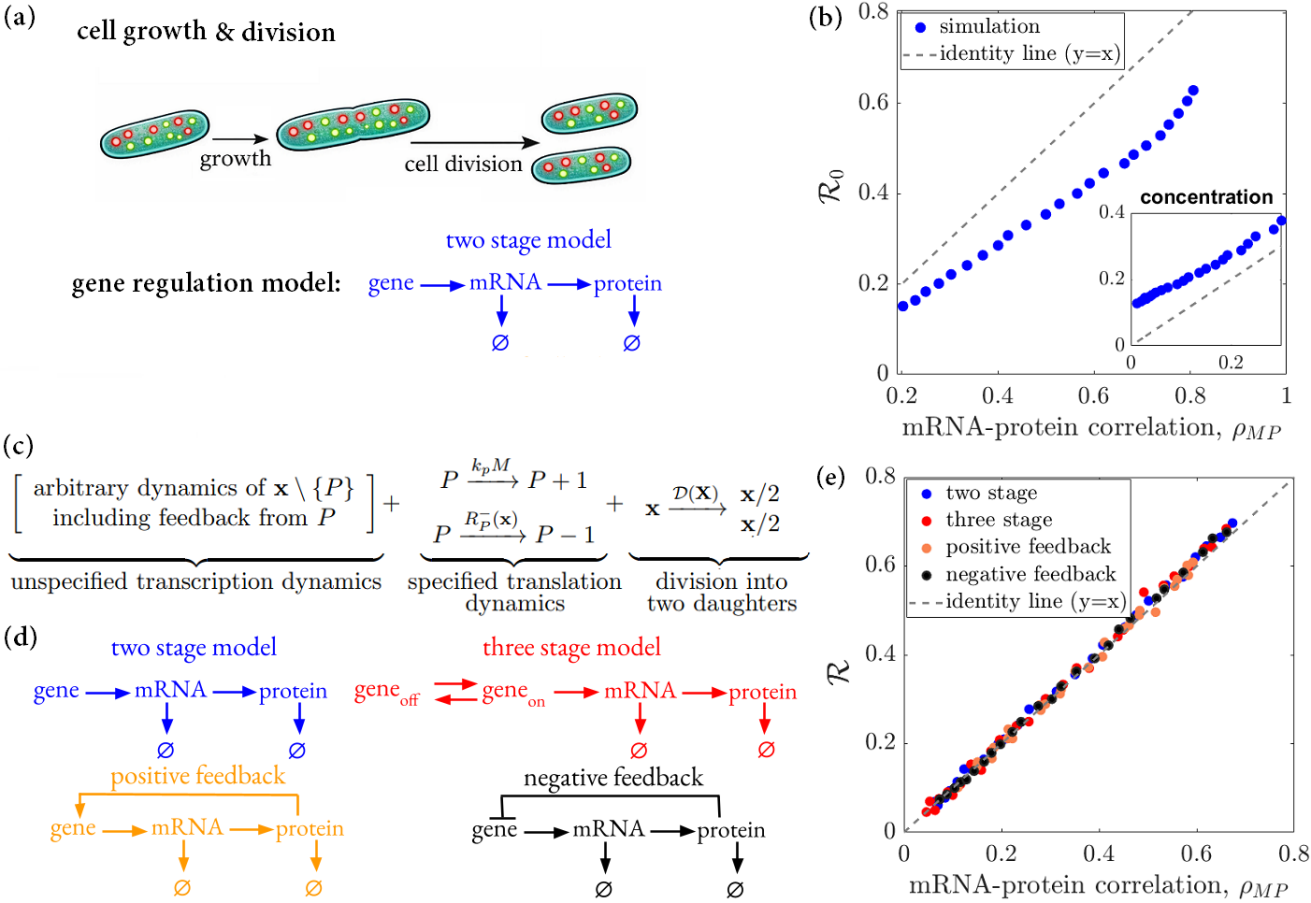
mRNA-protein correlation for growing and dividing cells. **(a)** Schematic of the growing-dividing model. Individual cells grow and divide into two daughter cells, while intracellular mRNA and protein copy numbers (yellow and red dots, respectively) evolve stochastically (top). The underlying gene-expression dynamics are represented by a two-stage regulatory module, in which a gene produces mRNA, which in turn produces protein, with both mRNA and protein undergoing degradation (bottom). **(b)** mRNA–protein correlation, $\rho_{MP}$, for growing-dividing cells deviates from the invariant prediction of Eq. (1). Inset **(b)**: the corresponding comparison for mRNA–protein concentration correlations, $\rho_{\left[M][P\right]}$. **(c)** For arbitrary dynamics of cellular components excluding proteins (x∖{P}), translation is modeled as a first-order process, with proteins produced at rate $k_{p}M$. Protein degradation is allowed to follow arbitrary kinetics, described by the flux $R_{𝒫}^{-}(\text{x})$, which may depend on the full cellular state. Upon division, all cellular components are partitioned between two daughter cells, with division occurring at a state-dependent rate 𝒟(x). **(d)** We consider several upstream transcription networks: a two-stage model (gene always active), a three-stage model (gene switches between active and inactive states at a constant rate) [25], and models with positive or negative feedback [26, 27]. For the two-stage and three-stage models, the mRNA production rate is $k_{m}g$ [25]. For positive and negative feedback, the mRNA production rate follows a Hill function [26, 27], $k_{m}g\frac{(P/K{)}^{n}}{1+(P/K{)}^{n}}$ and $k_{m}g\frac{1}{1+(P/K{)}^{n}}$, respectively, where K is the dissociation constant (half-maximal regulation) and n is the Hill coefficient (regulatory steepness). In all cases, the mRNA degradation rate is $\gamma_{m}M$. Definitions of model parameters are provided in Appendix Table 1. **(e)** Simulation results for $\rho_{MP}$ across the regulatory networks in (d) collapse onto the invariant prediction $\rho_{MP}=ℛ$ (Eq. 5), indicating universality with respect to upstream network architecture. In all panels, symbols denote stochastic simulations [28] of 10^3^ cells.

We consider a population in which each cell grows and divides into two daughter cells via binary fission, generating a branching lineage tree [18, 19] (see Fig. 2(a)). Within each cell, we consider the expression of a gene embedded in an arbitrary, unspecified network that affects transcription dynamics, while we specify the translation process (discussed in detail in the following sections). We make no assumptions about the growth process or the division-control mechanism. At division, cellular components are partitioned independently between the two daughter cells according to binomial statistics.

Within this framework, the probability density of the cell state, 𝒫(x,t) (for brevity, we sometimes omit the explicit time dependence below), is governed by the master equation

(2) $$\frac{d𝒫(x,t)}{dt}=\sum_{k}\left[r_{k}\left(x-d_{k}\right)𝒫\left(x-d_{k},t\right)-r_{k}(x)𝒫(x,t)\right]+\int \;dy[𝒦(x|y)+𝒦(y-x|y)]𝒟(y)𝒫(y,t)-𝒟(x)𝒫(x,t)-\lambda 𝒫(x,t).$$

Here, $r_{k}\left(x-d_{k}\right)𝒫\left(x-d_{k}\right)$ is the probability flux from state $x-d_{k}$ to state x, and 𝒟(x) is the state-dependent division rate. The sum runs over all possible transitions k, and each transition is represented by an integer vector $d_{k}$, which is the difference between two states. The quantity 𝒦(x∣y) denotes the binomial partition kernel, i.e., the conditional probability that a daughter cell is born in state x given that the mother cell divided in state y. The term -λ𝒫(x,t) accounts for the exponentially growing population with growth rate λ, such that ∫𝒫(x,t)dx=1. Using the master equation (2), we derive an invariant relation for mRNA–protein copy-number correlations.

Taking the steady state limit of Eq. (2), we obtain a relation for the correlation between the protein copy number P and its production rate Γ:

(3) $$\rho_{\Gamma P}=\frac{{\text{CV}}_{P}}{{\text{CV}}_{\Gamma }}\frac{1}{\left(\gamma_{p}+\lambda \right)}\left[\gamma_{p}\left(1-\frac{1}{{\text{CV}}_{P}^{2}\langle P\rangle }\right)+\frac{\lambda }{2}\left(1+\frac{1}{{\text{CV}}_{P}^{2}}\left(\frac{1}{2}\frac{{\left\langle P^{2}\right\rangle }_{𝒟}}{\langle P\rangle^{2}}-1-\frac{1}{\left\langle P\right\rangle }-\frac{1}{2}\frac{\langle P\rangle_{𝒟}}{\langle P\rangle^{2}}\right)\right)\right],$$

which is derived in Appendix Sec. A. Here $\gamma_{p}$ is the protein degradation rate, $\langle P\rangle_{𝒟}$ and ${\left\langle P^{2}\right\rangle }_{𝒟}$ are, respectively, the first and second moments of the protein copy number at the time of cell division. Unless stated otherwise, all statistics are computed over the population ensemble. Notably, as in Ref. [8], this result does not depend on assuming first-order protein degradation or any other specific degradation mechanism. Rather, we require only that proteins have a constant mean lifetime, equal to $1/\gamma_{p}$. By Little’s law [20], the mean rate of protein loss then satisfies $\left\langle R_{𝒫}^{-}(x)\right\rangle =\gamma_{p}\langle P\rangle$, which we use in the Appendix to derive Eq. (3). Eq. (3) also applies to populations with random removal of cells (e.g., chemostats [21] or turbidostats [22]) and to fixed-size-population turnover models such as Moran processes [23]. Furthermore, Eq. (3) is invariant in the sense that its validity does not depend on the specific mechanisms governing mRNA dynamics, protein production and degradation, genome replication, or division control. In the limit where protein degradation is negligible compared with growth-mediated dilution, $\gamma_{p}\ll \lambda$ [24], Eq. (3) is reduced to

(4) $$\rho_{\Gamma P}=\frac{{\text{CV}}_{P}}{{\text{CV}}_{\Gamma }}\frac{1}{2}\left[1+\frac{1}{{\text{CV}}_{P}^{2}}\left(\frac{1}{2}\frac{{\left\langle P^{2}\right\rangle }_{𝒟}}{\langle P\rangle^{2}}-1-\frac{1}{\left\langle P\right\rangle }-\frac{1}{2}\frac{\langle P\rangle_{𝒟}}{\langle P\rangle^{2}}\right)\right].$$

To relate this result to the mRNA–protein correlation, we must further specify what sets the total protein production rate, Γ.

### 3.3 mRNA-protein correlation for same-gene pairs

The protein production rate may depend on different factors, including mRNA abundance or ribosome availability [7, 29]. Before turning to a more detailed analysis, we consider here arguably the simplest translation model for the same-gene pair. Within this model, the protein production rate Γ is proportional to the number of mRNAs, M, and the proportionality coefficient is constant throughout the cell cycle (but may depend on the growth condition) [2, 5, 25]. Other translation models are discussed in Appendix Sec. C.

Combining this Γ∝M assumption with Eq. (4) yields

(5) $$\rho_{MP}=\frac{{\text{CV}}_{P}}{{\text{CV}}_{M}}\frac{1}{2}\left[1+\frac{1}{{\text{CV}}_{P}^{2}}\left(\frac{1}{2}\frac{{\left\langle P^{2}\right\rangle }_{𝒟}}{\langle P\rangle^{2}}-1-\frac{1}{\left\langle P\right\rangle }-\frac{1}{2}\frac{\langle P\rangle_{𝒟}}{\langle P\rangle^{2}}\right)\right]\equiv ℛ.$$

We test this mRNA–protein correlation using the Gillespie simulation [28] across several gene circuits (Fig. 2(d)). In these simulations, cells grow and divide deterministically, with stochastic binomial partitioning of cellular components at division. As shown in Fig. 2(e), Eq. (5) agrees with the stochastic simulations for all scenarios considered. The last term, $-\frac{1}{2}\frac{\langle P\rangle_{𝒟}}{\langle P\rangle^{2}}$, in Eq. (5) accounts for binomial partitioning at cell division; omitting this term reduces the equation to the corresponding result for deterministic equal partitioning. At large copy numbers, the two predictions are indistinguishable (Appendix Fig. A.1 (b) for ⟨P⟩≃100), indicating that the effect of binomial partitioning is negligible in this limit. At low copy numbers, however, the deterministic-equal-partitioning approximation deviates from the binomial-partitioning result as expected (Appendix Fig. A.1 (a) for ⟨P⟩≃10).

Under the same assumption Γ∝M and in the limit λ→0, with a finite mean protein lifetime $1/\gamma_{p}$, Eq. (3) reduces to Eq. (1), thereby recovering the expression previously derived for mRNA–protein correlations in constant-volume, non-dividing cells [8]. In contrast to Eq. (5), Eq. (1) fails to predict the mRNA–protein copy numbers and concentration correlations in growing and dividing cells (see Fig. 2(b)).

### 3.4 mRNA-protein correlation for different-gene pairs

For different-gene pairs, if mRNA and protein neither regulate one another (not even indirectly) nor share upstream regulation, and if cell volume is their sole common source of correlation, then the correlation between M and P is given by

(6) $$\rho_{MP}=\rho_{VM}\;\rho_{VP},$$

provided that both variables depend linearly on cell volume, V, with residual fluctuations that are uncorrelated with one another and with V [30]. Here, $\rho_{VM}$ and $\rho_{VP}$ denote the mRNA–volume and protein–volume correlations, respectively. We compare Eq. (6) for different-gene pairs against stochastic simulations and find good agreement (see Appendix Fig. E.1).

### 3.5 Comparison with experimental data

**Figure 3:**
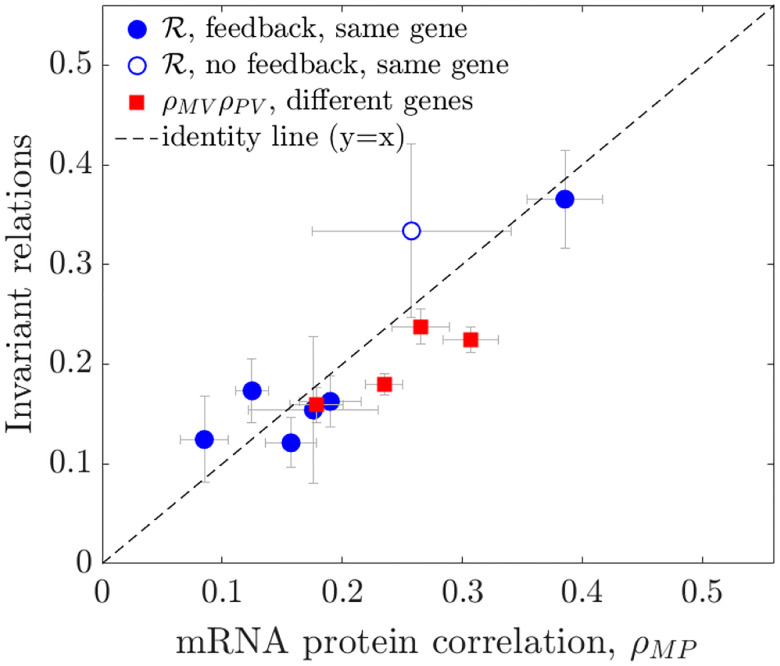
Experimental validation of mRNA–protein correlations predicted by Eq. (5) and (6). For the same experiments introduced in Sec. 3.1, mRNA–protein copy number correlations for different-gene pairs (red squares) are well described by Eq. (6), whereas Eq. (5) accurately reproduces the measured correlations for same-gene mRNA–protein pairs (blue circles). Error bars indicate bootstrap standard deviations obtained by resampling individual cells. The dashed line indicates y=x (perfect agreement between theory and experiment). Estimating ℛ from Eq. (5) requires $\langle P\rangle_{𝒟}$ and ${\left\langle P^{2}\right\rangle }_{𝒟}$, which cannot be obtained directly from the experimental data. We infer them from the protein time series using the Bayesian procedure described in Appendix Sec. B, and estimate the remaining mRNA/protein moments directly from the data.

We test the predicted mRNA–protein correlations against the single-cell *E. coli* measurements [11] introduced in Sec. (3.1). For same-gene pairs, the measured mRNA–protein correlations, both with and without feedback, are consistent with the invariant prediction of Eq. (5) (blue circles in Fig. 3). None of these points deviates significantly from the identity line; after Bonferroni correction, all p-values are equal to 1. For different-gene pairs, the measured correlations are likewise consistent with the volume-mediated prediction of Eq. (6) (red squares in Fig. 3(b)). Again, none of these points deviates significantly from the identity line, with all Bonferroni-corrected p-values exceeding 0.05.

To further examine the key assumption behind Eq. (6)—namely, that cell volume is the only confounder for different-gene pairs—we compute correlations between mRNA and protein *concentrations* (Appendix Fig. D.1). As expected, for same-gene pairs, the concentration correlations remain positive; whereas for different-gene pairs, the concentration correlations drop markedly. For two of the conditions, their value are statistically indistinguishable from zero (p>0.1). For the other two, it is significantly positive $\left(p<10^{-6}\right)$, indicating that cell volume is not the only confounding factor, at least for these two conditions (see Appendix Fig. D.1).

So far, we have adopted the simplifying assumption that the protein production rate is proportional to mRNA copy number, Γ∝M, leading to Eq. (5). More generally, translation may be limited by other factors, such as ribosome availability and mRNA fraction in the total mRNA pool, while transcription may be limited by gene copy number or RNAP copy number and gene fraction, giving rise to distinct gene-expression regimes [7, 29], as discussed in detail in Appendix Sec. C. We find that under fast-growth conditions, Eq. (5) holds in all regimes. Under slow-growth conditions, however, it is expected to fail in one regime, where transcription is limited solely by gene copy number, and translation is limited by both ribosome availability and mRNA fraction. However, the experimental data in Ref. [11] were collected in fast growth conditions, where the approximation Γ∝M, hence, Eq. (5) is also expected to hold. Discriminating among translation-limited regimes using single-cell mRNA-protein data, therefore, requires measurements at slower growth conditions.

### 3.6 Measurement noise

In addition to intrinsic noise, measurement noise can induce an additional source of variability in mRNA–protein correlations that was not included in the preceding analysis. Incorporating measurement noise yields a modified invariant relation for the correlation between the measured mRNA abundance, $\overset{ˆ}{M}$, and the measured protein abundance, $\overset{ˆ}{P}$, namely $\rho_{\overset{ˆ}{M}\overset{ˆ}{P}}=\overset{ˆ}{ℛ}$ (see Eq. (A.33) in Appendix Sec. A.3).

Importantly, although mRNA measurement noise reduces the observed mRNA–protein correlation, it does not alter the invariant relation in Eq. (5), because Eq. (A.33) reduces to Eq. (5) in the absence of protein measurement noise (see Appendix Sec. A.3). As shown in Appendix Fig. A.2(a), multiplicative measurement noise in protein copy number reduces the observed correlation $\rho_{\overset{ˆ}{M}\overset{ˆ}{P}}$ relative to the noiseless prediction of Eq. (5), thereby generating a systematic deviation from that relation. Nevertheless, Eq. (A.33) accurately predicts the correlation over a broad range of protein measurement noise levels, as illustrated in Appendix Fig. A.2(b).

### 3.7 Calibration of protein copy number

Absolute quantification of protein copy number in single cells remains technically challenging and depends critically on accurate calibration [31]. Importantly, Eq. (5) is not invariant under a rescaling of P. If the true protein copy number P is related to the measured, uncalibrated signal $\overset{ˆ}{P}$ by $P=f\overset{ˆ}{P}$, where f is a constant calibration factor, then this rescaling leaves the mRNA–protein correlation coefficient $\rho_{MP}$ unchanged. By contrast, the theoretical prediction ℛ in Eq. (5) depends explicitly on f through terms proportional to 1/⟨P⟩ and $\langle P\rangle_{𝒟}/\langle P\rangle^{2}$. Consequently, an incorrect calibration factor introduces a systematic discrepancy between the predicted and observed correlations. This sensitivity enables Eq. (5) to be used to infer the protein calibration factor.

To quantify the agreement between theory and data (simulation and experiment), we compute, for each value of f, the root-mean-square (RMS) error, $\sqrt{\left\langle d_{i}^{2}\right\rangle }$, where $d_{i}=ℛ-\rho_{MP}$ is the residual for data point i, defined as the difference between the theoretical prediction and the measured correlation. The calibration factor is then inferred as the value $f_{\text{min}}$ that minimizes the RMS error. To estimate the uncertainty in $f_{\text{min}}$, we performed bootstrap resampling of the individual sample points. We used the resulting distribution of $f_{\text{min}}$ to determine its 95% bootstrap confidence interval, shown as an error bar in Fig. A.3.

Applying this procedure to simulated data yields $f_{\text{min}}=1.07$, with a 95% confidence interval of 0.92–1.30, as expected for correctly calibrated protein copy numbers (Appendix Fig. A.3(a)). However, for *E. coli* data [11] we obtain $f_{\text{min}}=0.52$, with a substantially broader 95% confidence interval of 0.36–1.07 (Appendix Fig. A.3(b)). This broader interval likely reflects both the limited number of data points (n=7) and their relatively large uncertainty, suggesting that additional measurements and improved precision should substantially sharpen the estimate of f. Moreover, the value of f that we find here falls within the estimated error of the calibration procedure in [11].

### 3.8 Invariant relation in a mother-machine setup

So far, we have considered a population in which each division produces two daughter cells that both remain in the population. We now contrast this with a *lineage* setting, motivated by mothermachine experiments: a single “mother” cell is trapped at the closed end of a growth channel, while its newborn progeny are displaced by growth and washed out, enabling long-term tracking of a single lineage [32]. For this lineage ensemble, we obtain the following invariant relation (see Appendix Sec. F):

(7) $$\rho_{\Gamma P}=\frac{1}{{\text{CV}}_{\Gamma }{\text{CV}}_{P}}\frac{\frac{3}{4}{\left\langle P^{2}\right\rangle }_{𝒟}-\langle P\rangle_{𝒟}\langle P\rangle -\langle P\rangle_{𝒟}}{\left\langle P\rangle_{𝒟}\langle P\right\rangle },$$

which differs from the invariant relation in Eq. (4), derived for the scenario where both daughter cells remain after division in the population or other systems with random cell dilution. This difference indicates that the invariant relation is ensemble-dependent, reflecting the distinct age structure and sampling statistics that affect mRNA–protein correlations.

To connect Eq. (7) to mRNA–protein correlations, we specify the total protein production rate Γ for the three gene-expression regimes discussed in Appendix Sec. C. The qualitative picture remains unchanged. Under fast-growth conditions, all three regimes become effectively indistinguishable and collapse onto the same invariant relation (Appendix Fig. F.1(a)). For slow-growth conditions, in the regime where transcription is limited by gene copy number only, and translation is limited by both ribosomes and mRNA fraction, a distinct relation is valid (see Appendix Sec. F).

## 4 Discussion

Quantitative models of complex biological systems often involve many interacting components with poorly constrained parameters [33]. This “curse of dimensionality” can increase the risk of overfitting empirical data [34]. An alternative strategy is to focus on a subsystem of interest, introducing simplifying assumptions about the remaining components and testing the robustness of the resulting conclusions to these assumptions [33]. Only in rare cases can exact invariant relations be derived—relations that depend solely on the subsystem of interest and remain insensitive to the details of the rest of the system [8]. An analogous situation arises in statistical mechanics, where a small number of universal relations, such as the Jarzynski equality [35], apply to broad classes of nonequilibrium systems despite widely differing microscopic dynamics.

In this work, we extended the framework of Ref. [8] to growing and dividing cells and derived invariant relations connecting the covariance between an mRNA and its protein product to their first and second moments. A key result is that these relations depend only on the details of translation and remain insensitive to the potentially complex upstream transcriptional dynamics. At the same time, we showed that cellular growth and division qualitatively modify the form of these invariants. In particular, we obtained distinct results for *(i)* populations of cells with constant size, which recover the results of Ref. [8]; *(ii)* growing and dividing cells subject either to no removal or to stochastic removal of cells at division; and *(iii)* growing and dividing cells in which one daughter cell is always removed, as in mother-machine experiments [32].

Throughout, we considered a translation model in which the protein production rate scales linearly with mRNA abundance across all regimes examined in Sec. C. Although this assumption is widely used [2, 5, 7, 25], it is not universally valid; for example, a finite ribosome pool may produce a sublinear, Michaelis–Menten-like dependence of protein production on mRNA levels [36]. By analyzing single-gene measurements using the invariant relation derived here, we find that the linear approximation is consistent with the available empirical data, while noting that it need not hold for other genes, organisms, or growth conditions. Finally, our analysis focused on relations between the moments of absolute mRNA and protein copy numbers. In many contexts, however, concentrations are the more relevant observables. Unlike systems of constant volume, for growing and dividing cells, the statistics of concentrations can differ substantially from those of absolute copy numbers. Likewise, conditioning on cell volume may strongly reduce, or even eliminate, apparent correlations between mRNA and protein abundances, as in Ref. [37]. Extending the present framework to more general settings, including concentration-based statistics and volume-conditioned correlations, is therefore a natural direction for future work.

Recent advances in fluorescent protein labeling [38] and mRNA quantification technique [39, 40, 41], combined with the invariant relations derived here, may provide a useful framework for addressing quantitative aspects of the central dogma. Because these relations hold under broad conditions and depend only on the effective translation dynamics, they can serve as consistency checks for synthetic genetic circuits and experimental measurement pipelines. In particular, deviations from the predicted invariant relations may indicate translation dynamics in which protein production is not proportional to mRNA copy number or non-binary fission, neither of which is incorporated into the present analysis. Moreover, as demonstrated in Sections 3.6 and 3.7, the predicted invariant relation can be leveraged to estimate measurement noise and to calibrate experimental readouts, thereby enabling the conversion of fluorescence intensity into absolute protein copy numbers. More generally, our results provide a principled approach for probing translation dynamics within arbitrary transcriptional circuits without requiring detailed knowledge of the upstream regulatory architecture. In this sense, the framework developed here may be viewed not only as a tool for studying mRNA–protein coupling, but also as a more general strategy for isolating and characterizing a reaction of interest embedded in a complex and only partially observed interaction network in growing and dividing cells.

## Supplementary Material

1
