# Supplementary material for: Cell Growth and Division Shape mRNA–Protein Correlations
Source: bioRxiv. 2026 May 6:2026.05.04.722628. Preprint. [Version 1] doi: 10.64898/2026.05.04.722628 (PMC13174372; doi:10.64898/2026.05.04.722628)
Supplement: 1 [file NIHPP2026.05.04.722628V1-supplement-1.pdf]

bioRxiv preprint doi: https://doi.org/10.64898/2026.05.04.722628; this version posted May 6, 2026. The copyright holder for this preprint (which was not certified by peer review) is the author/funder, who has granted bioRxiv a license to display the preprint in perpetuity. It is made available under aCC-BY 4.0 International license.

A Calculation of invariant relations for a population of growing and dividing cells

Here we derive Eqs. (3) and (F.15) building on the formalism developed in [8]. As mentioned in the main text, we consider a population of growing and dividing cells into two daughter cells, forming a tree with or without random cell dilution. Within the cells, we consider the expression of a gene embedded in an arbitrary, unspecified network that affects transcription dynamics, while we specify the translation process [8]. We make no assumptions about the growth process or the division-control mechanism. We start from the Markovian master equation for the probability density of the cell state described by the vector of numbers of all its components

$$\begin{aligned}\frac{d\mathcal{P}(\mathbf{x},t)}{dt} &= \sum_k \left[r_k(\mathbf{x}-\mathbf{d}_k)\mathcal{P}(\mathbf{x}-\mathbf{d}_k) - r_k(\mathbf{x})\mathcal{P}(\mathbf{x})\right] \\ &\quad + \int d\mathbf{y}\left[\mathcal{K}(\mathbf{x}|\mathbf{y}) + \mathcal{K}(\mathbf{y}-\mathbf{x}|\mathbf{y})\right] - \mathcal{D}(\mathbf{x})\mathcal{P}(\mathbf{x}) - \lambda\mathcal{P}(\mathbf{x}),\end{aligned} \tag{A.1}$$

where we assume binomial partitioning of cell components

$$\mathcal{K}(\mathbf{x}|\mathbf{y}) = \prod_i \mathrm{Bin}(x_i; y_i, 1/2) = \prod_i \binom{y_i}{x_i}\frac{1}{2^{y_i}}. \tag{A.2}$$

Previously mentioned in the main text, $r_k(\mathbf{x}-\mathbf{d}_k)\mathcal{P}(\mathbf{x}-\mathbf{d}_k)$ is the probability flux from state $\mathbf{x}-\mathbf{d}_k$ to state $\mathbf{x}$ and $\mathcal{D}(\mathbf{x})$ is the division rate at state $\mathbf{x}$. For the deterministic scenario, $\mathcal{D}(\mathbf{x})$ will be a delta function. The sum runs over all possible transitions k and each transition is represented by an integer vector $\mathbf{d}_k$. To keep the normalization $\int \mathcal{P}(\mathbf{x},t)d\mathbf{x} = 1$ we remove random cells from the population with the growth rate λ, resulting in the $-\lambda\mathcal{P}(\mathbf{x})$ term.

The population averages $\langle x_i\rangle(t) = \int \mathcal{P}(\mathbf{x},t)x_i d\mathbf{x}$ follow

$$\frac{d\langle x_i\rangle}{dt} = \left\langle \sum_k d_{ik}r_k(\mathbf{x})\right\rangle + \langle y_i\mathcal{D}(\mathbf{y})\rangle - \langle x_i\mathcal{D}(\mathbf{x})\rangle - \lambda\langle x_i\rangle \tag{A.3}$$

$$= \left\langle \sum_k d_{ik}r_k(\mathbf{x})\right\rangle - \lambda\langle x_i\rangle = \left\langle R_i^+(\mathbf{x})\right\rangle - \left\langle R_i^-(\mathbf{x})\right\rangle - \lambda\langle x_i\rangle, \tag{A.4}$$

where we used $\mathcal{K}(\mathbf{x}|\mathbf{y}) = \mathcal{K}(\mathbf{y}-\mathbf{x}|\mathbf{y})$, $\langle x_i\mathcal{K}(\mathbf{x}|\mathbf{y})\rangle = y_i/2$ and

$$R_i^+ = \sum_{k:d_{ik}>0} d_{ik}r_k(\mathbf{x}) \text{ and } R_i^- = \sum_{k:d_{ik}<0} |d_{ik}|r_k(\mathbf{x}). \tag{A.5}$$

Here d_{ik} is the i'th element of the $\mathbf{d}_k$ vector. At the stationarity, $\frac{d\langle x_i\rangle}{dt} = 0$ we have a bias towards the positive rate to balance the cell divisions:

$$\left\langle R_i^+\right\rangle - \left\langle R_i^-\right\rangle = \lambda\langle x_i\rangle. \tag{A.6}$$

To calculate the second moments we use

$$\langle x_i x_j\mathcal{K}(\mathbf{x}|\mathbf{y})\rangle = \frac{y_i y_j}{4} + \delta_{ij}\frac{y_i}{4} \tag{A.7}$$

bioRxiv preprint doi: https://doi.org/10.64898/2026.05.04.722628; this version posted May 6, 2026. The copyright holder for this preprint (which was not certified by peer review) is the author/funder, who has granted bioRxiv a license to display the preprint in perpetuity. It is made available under aCC-BY 4.0 International license.

and get

$$\frac{d\langle x_ix_j\rangle}{dt} = \sum_k \left[d_{ik}d_{jk}\langle r_k(\mathbf{x})\rangle + d_{jk}\langle x_ir_k(\mathbf{x})\rangle + d_{ik}\langle x_jr_k(\mathbf{x})\rangle\right] - \frac{\lambda}{2}\langle x_ix_j\rangle_{\mathcal{D}} + \frac{\lambda}{2}\delta_{ij}\langle x_i\rangle_{\mathcal{D}} - \lambda\langle x_ix_j\rangle,$$
$$= \sum_k d_{ik}d_{jk}\langle r_k(\mathbf{x})\rangle + \left\langle x_i(R_j^+ - R_j^-)\right\rangle + \left\langle x_j(R_i^+ - R_i^-)\right\rangle - \frac{\lambda}{2}\langle x_ix_j\rangle_{\mathcal{D}} + \frac{\lambda}{2}\delta_{ij}\langle x_i\rangle_{\mathcal{D}} - \lambda\langle x_ix_j\rangle,$$
$$= B_{ij} + \left\langle (x_i - \langle x_i\rangle)(R_j^+ - R_j^-)\right\rangle + \lambda\langle x_j\rangle\langle x_i\rangle$$
$$+ \left\langle (x_j - \langle x_j\rangle)(R_i^+ - R_i^-)\right\rangle + \lambda\langle x_j\rangle\langle x_i\rangle - \frac{\lambda}{2}\langle x_ix_j\rangle_{\mathcal{D}} + \frac{\lambda}{2}\delta_{ij}\langle x_i\rangle_{\mathcal{D}} - \lambda\langle x_ix_j\rangle,$$
$$= B_{ij} + \mathrm{Cov}\left(x_j, R_i^+\right) - \mathrm{Cov}\left(x_j, R_i^-\right) + \mathrm{Cov}\left(x_i, R_j^+\right) - \mathrm{Cov}\left(x_i, R_j^-\right)$$
$$+ 2\lambda\langle x_j\rangle\langle x_i\rangle - \frac{\lambda}{2}\langle x_ix_j\rangle_{\mathcal{D}} + \frac{\lambda}{2}\delta_{ij}\langle x_i\rangle_{\mathcal{D}} - \lambda\langle x_ix_j\rangle = 0, \tag{A.8}$$

where

$$\langle x_ix_j\rangle_{\mathcal{D}} = \frac{\int x_ix_j\mathcal{D}(\mathbf{x})\mathcal{P}(\mathbf{x})\,d\mathbf{x}}{\int \mathcal{D}(\mathbf{x})\mathcal{P}(\mathbf{x})\,d\mathbf{x}} = \frac{\langle x_ix_j\mathcal{D}(\mathbf{x})\rangle}{\langle\mathcal{D}(\mathbf{x})\rangle} \tag{A.9}$$

denotes the average value of x_ix_j at division. The normalization $\int \mathcal{D}(\mathbf{x})\mathcal{P}(\mathbf{x})\,d\mathbf{x} = \langle\mathcal{D}(\mathbf{x})\rangle = \lambda$ corresponds to the population growth rate. With this definition, the contribution $-\frac{\lambda}{2}\langle x_ix_j\rangle_{\mathcal{D}}$ in Eq. (A.8) arises from the term $\langle 2x_ix_j\mathcal{D}(2\mathbf{x})\rangle - \langle x_ix_j\mathcal{D}(\mathbf{x})\rangle$.

The diffusion matrix can be written as

$$B_{ij} = \sum_k d_{ik}d_{jk}\langle r_k(x)\rangle = \sum_k |d_{ik}|\,|d_{jk}|\,\mathrm{sgn}(d_{ik}d_{jk})\langle r_k(x)\rangle, \tag{A.10}$$
$$= \sum_k \underbrace{\left(\frac{|d_{ik}|\,\langle r_k(x)\rangle}{\sum_l |d_{il}|\,\langle r_l(x)\rangle}\right)}_{\rho_{ik}} |d_{jk}|\,\mathrm{sgn}(d_{ik}d_{jk}) \underbrace{\sum_l |d_{il}|\,\langle r_l(x)\rangle}_{\langle R_i^+ + R_i^-\rangle}, \tag{A.11}$$
$$= \langle s_{ij}\rangle\left\langle R_i^+ + R_i^-\right\rangle, \tag{A.12}$$

by defining

$$\langle s_{ij}\rangle = \sum_k \rho_{ik}|d_{jk}|\,\mathrm{sgn}(d_{ik}d_{jk}). \tag{A.13}$$

In sum, we get

$$\langle s_{ij}\rangle\left\langle R_i^+ + R_i^-\right\rangle + \mathrm{Cov}\left(x_j, R_i^+\right) - \mathrm{Cov}\left(x_j, R_i^-\right) + \mathrm{Cov}\left(x_i, R_j^+\right) - \mathrm{Cov}\left(x_i, R_j^-\right)$$
$$+ 2\lambda\langle x_j\rangle\langle x_i\rangle - \frac{\lambda}{2}\langle x_ix_j\rangle_{\mathcal{D}} + \frac{\lambda}{2}\delta_{ij}\langle x_i\rangle_{\mathcal{D}} - \lambda\langle x_ix_j\rangle = 0. \tag{A.14}$$

Using

$$\left\langle R_i^+\right\rangle - \left\langle R_i^-\right\rangle = \lambda\langle x_i\rangle, \tag{A.15}$$

one gets

$$\langle s_{ij}\rangle\left(\lambda\langle x_i\rangle + 2\left\langle R_i^-\right\rangle\right) + \mathrm{Cov}\left(x_j, R_i^+\right) - \mathrm{Cov}\left(x_j, R_i^-\right) + \mathrm{Cov}\left(x_i, R_j^+\right) - \mathrm{Cov}\left(x_i, R_j^-\right)$$
$$+ 2\lambda\langle x_j\rangle\langle x_i\rangle - \frac{\lambda}{2}\langle x_ix_j\rangle_{\mathcal{D}} + \frac{\lambda}{2}\delta_{ij}\langle x_i\rangle_{\mathcal{D}} - \lambda\langle x_ix_j\rangle = 0. \tag{A.16}$$

bioRxiv preprint doi: https://doi.org/10.64898/2026.05.04.722628; this version posted May 6, 2026. The copyright holder for this preprint (which was not certified by peer review) is the author/funder, who has granted bioRxiv a license to display the preprint in perpetuity. It is made available under aCC-BY 4.0 International license.

Assuming that all processes except division change the number of all elements by ± 1 we get $\langle s_{ij} \rangle = 1$. Denoting average degradation time of x_i as τ_i and using Little's law [20] we get $\langle R_i^- \rangle = \langle x_i \rangle / \tau_i$. This results in

$$\left(\lambda \langle x_i \rangle + \frac{2 \langle x_i \rangle}{\tau_i} \right) + \mathrm{Cov}\left(x_j, R_i^+ - R_i^-\right) + \mathrm{Cov}\left(x_i, R_j^+ - R_j^-\right) + 2\lambda \langle x_j \rangle \langle x_i \rangle - \frac{\lambda}{2} \langle x_i x_j \rangle_{\mathcal{D}} + \frac{\lambda}{2} \delta_{ij} \langle x_i \rangle_{\mathcal{D}} - \lambda \langle x_i x_j \rangle = 0 \quad (A.17)$$

$$\mathrm{Cov}\left(x_j, R_i^+ - R_i^-\right) + \mathrm{Cov}\left(x_i, R_j^+ - R_j^-\right) \quad (A.18)$$

$$= -2\frac{\langle x_i \rangle}{\tau_i} - \lambda \left(\langle x_i \rangle + \langle x_j \rangle \langle x_i \rangle - \frac{1}{2} \langle x_i x_j \rangle_{\mathcal{D}} + \frac{1}{2} \delta_{ij} \langle x_i \rangle_{\mathcal{D}} - \mathrm{Cov}(x_i, x_j) \right) \quad (A.19)$$

For $i = j$ we get

$$\mathrm{Cov}\left(x_i, R_i^+\right) = \frac{\mathrm{Var}\left(x_i\right) - \langle x_i \rangle}{\tau_i} + \frac{\lambda}{2} \left(\frac{\langle x_i^2 \rangle_{\mathcal{D}}}{2} + \mathrm{Var}(x_i) - \langle x_i \rangle^2 - \langle x_i \rangle - \frac{\langle x_i \rangle_{\mathcal{D}}}{2} \right). \quad (A.20)$$

Consider now a more specific situation where index 1 denotes mRNA, $x_1 = M$, and index 2 denotes protein translated from this mRNA, $x_2 = P$. Assuming a constant protein degradation rate γ_p and some M dependent protein production rate $R_2^+ = \mathit{\Gamma}$ we have

$$\underbrace{\begin{bmatrix} \text{arbitrary dynamics of } M, x_3, x_4, \dots \\ \text{including feedback loops} \end{bmatrix}}_{\substack{\text{unspecified} \\ \text{network dynamics}}} + \underbrace{\begin{matrix} P \xrightarrow{\mathit{\Gamma}} P+1 \\ P \xrightarrow{\gamma_p P} P-1 \end{matrix}}_{\substack{\text{specified} \\ P\text{-dynamics}}} \quad (A.21)$$

From Eq. (A.15), substituting $i = 2$ and $x_2 = P$, we get

$$\langle \mathit{\Gamma} \rangle - \gamma_p \langle P \rangle = \lambda \langle P \rangle . \quad (A.22)$$

Using this, we get the correlation between the protein copy number P and the total protein production rate $\mathit{\Gamma}$ as follows

$$\rho_{\mathit{\Gamma}P} = \frac{\mathrm{Cov}\left(P, \mathit{\Gamma}\right)}{\mathrm{CV}_P \mathrm{CV}_{\mathit{\Gamma}} \langle P \rangle \langle \mathit{\Gamma} \rangle} = \frac{\mathrm{CV}_P}{\mathrm{CV}_{\mathit{\Gamma}}} \frac{1}{(\gamma_p + \lambda)} \left\{ \gamma_p \left(1 - \frac{1}{\mathrm{CV}_P^2 \langle P \rangle} \right) + \frac{\lambda}{2} \left[1 + \frac{1}{\mathrm{CV}_P^2} \left(\frac{1}{2} \frac{\langle P^2 \rangle_{\mathcal{D}}}{\langle P \rangle^2} - 1 - \frac{1}{\langle P \rangle} - \frac{1}{2} \frac{\langle P \rangle_{\mathcal{D}}}{\langle P \rangle^2} \right) \right] \right\}. \quad (A.23)$$

For $\gamma_p \ll \lambda$, Eq. (A.23) simplified to

$$\rho_{\mathit{\Gamma}P} = \frac{\mathrm{CV}_P}{\mathrm{CV}_{\mathit{\Gamma}}} \frac{1}{2} \left[1 + \frac{1}{\mathrm{CV}_P^2} \left(\frac{1}{2} \frac{\langle P^2 \rangle_{\mathcal{D}}}{\langle P \rangle^2} - 1 - \frac{1}{\langle P \rangle} - \frac{1}{2} \frac{\langle P \rangle_{\mathcal{D}}}{\langle P \rangle^2} \right) \right], \quad (A.24)$$

which is the same as Eq. (4) in the main text. Eq. (A.24) is invariant in the sense that it does not depend on the transcription, translation, degradation, genome replication, or division control. Next, we will discuss different forms of invariant relation in Eq. (A.24) for different scenarios of protein production rate $\mathit{\Gamma}$.

bioRxiv preprint doi: https://doi.org/10.64898/2026.05.04.722628; this version posted May 6, 2026. The copyright holder for this preprint (which was not certified by peer review) is the author/funder, who has granted bioRxiv a license to display the preprint in perpetuity. It is made available under aCC-BY 4.0 International license.

A.1 Invariant relation for mRNA copy number limited translation

If the protein production rate Γ is proportional to the number of mRNAs, M, one gets the following dynamics

$$\underbrace{\begin{bmatrix} \text{arbitrary dynamics of } M, x_3, x_4, \ldots \\ \text{including feedback loops} \end{bmatrix}}_{\substack{\text{unspecified} \\ \text{network dynamics}}} + \underbrace{\begin{matrix} P \xrightarrow{\Gamma = k_p M} P+1 \\ P \xrightarrow{\gamma_p P} P-1 \end{matrix}}_{\substack{\text{specified} \\ X_2\text{-dynamics}}} \tag{A.25}$$

Using Eq. (A.24) with $\Gamma \propto M$ we obtain

$$\rho_{MP} = \frac{\mathrm{CV}_P}{\mathrm{CV}_M}\frac{1}{2}\left[1 + \frac{1}{\mathrm{CV}_P^2}\left(\frac{1}{2}\frac{\langle P^2\rangle_{\mathcal{D}}}{\langle P\rangle^2} - 1 - \frac{1}{\langle P\rangle} - \frac{1}{2}\frac{\langle P\rangle_{\mathcal{D}}}{\langle P\rangle^2}\right)\right] \equiv \mathcal{R}, \tag{A.26}$$

which is the same mRNA–protein correlation in the main text in Eq. (5).

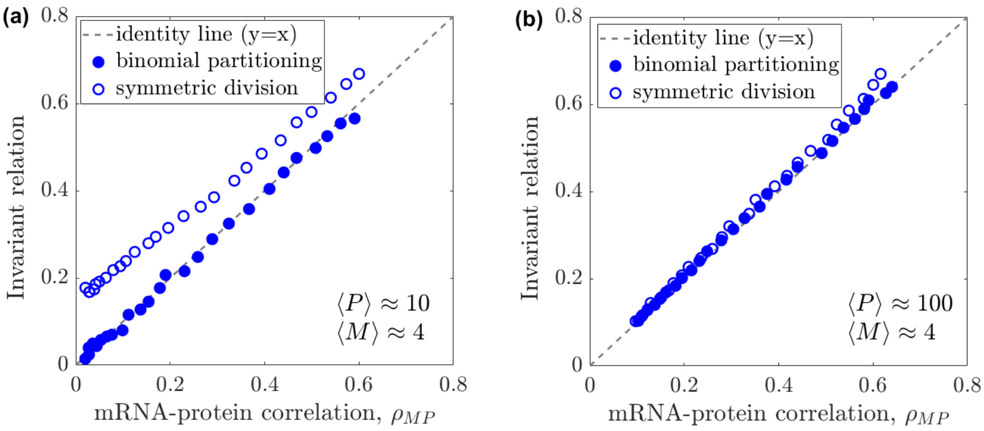

Figure A.1: **mRNA-protein correlation for symmetric and binomial partitioning.** The mRNA–protein correlation, ρ_{MP}, obtained from stochastic simulations with binomial partitioning at cell division, is plotted against the corresponding invariant prediction. Filled symbols show the prediction of Eq. (A.26) including the binomial-partitioning correction, whereas open symbols show the corresponding prediction obtained by omitting the last term, $-\frac{1}{2}\frac{\langle P\rangle_{\mathcal{D}}}{\langle P\rangle^2}$ in Eq. (A.26), i.e., the approximation for deterministic equal partitioning at division. **(a)** In the low-copy-number regime, the full invariant prediction, including binomial partitioning, remains in good agreement with the simulation results, whereas the deterministic-equal-partitioning approximation shows a clear deviation. **(b)** In the high-copy-number regime, the two predictions become nearly indistinguishable, showing that the correction due to binomial partitioning becomes negligible at large copy numbers. The symbols are obtained from stochastic simulation [28] over 10^3 cells.

Stochastic binomial partitioning at cell division reduces correlations between cellular components relative to deterministic symmetric division. This reduction is set by the variance of the binomial distribution and is captured by the $\delta_{ij}\frac{y_i}{4}$ term in Eq. (A.7). In turn, this contribution generates the $-\frac{1}{2}\frac{\langle P\rangle_{\mathcal{D}}}{\langle P\rangle^2}$ terms in Eqs. (A.23,A.24,A.26). Omitting this term, one obtains the expressions corresponding to deterministic symmetric cell division. As shown in Fig. A.1(b), the predicted correlations for binomial and symmetric partitioning become indistinguishable at large protein copy numbers, indicating that the correction due to binomial partitioning is negligible in this limit. At low protein copy numbers, however, the symmetric division deviates from the binomial-partitioning result as expected (Fig. A.1(a)).

bioRxiv preprint doi: https://doi.org/10.64898/2026.05.04.722628; this version posted May 6, 2026. The copyright holder for this preprint (which was not certified by peer review) is the author/funder, who has granted bioRxiv a license to display the preprint in perpetuity. It is made available under aCC-BY 4.0 International license.

A.2 Invariant relation for ribosome and mRNA fraction limited translation

If the protein production rate is limited by the ribosome number and mRNA fraction, then $\Gamma \propto \frac{RM}{\sum_j M_j} \propto \frac{RM}{\sum_j g_j}$, if the transcription rate is limited by the total gene dosage, such that $\sum_j M_j \propto \sum_j g_j$. The denominator, $\sum_j g_j$, scales with the total cellular DNA content $DNA(t)$, which increases piecewise linearly and doubles once per cell cycle [42, 43]. Assuming that the number of ribosomes scales linearly with the volume $R \propto V(t)$, one gets the following dynamics

$$\underbrace{\left[\begin{array}{c}\text{arbitrary dynamics of } M, x_3, x_4, \dots \\ \text{including feedback loops}\end{array}\right]}_{\substack{\text{unspecified}\\ \text{network dynamics}}} + \underbrace{\begin{array}{c} P \xrightarrow{\Gamma \propto \frac{V(t)M(t)}{DNA(t)}} P+1 \\ P \xrightarrow{\gamma_P P} P-1 \end{array}}_{\substack{\text{specified}\\ P\text{-dynamics}}} \tag{A.27}$$

Using Eq. (A.24) with $\Gamma \propto \widetilde{M}$, by defining $MV/DNA = \widetilde{M}$, we obtain

$$\rho_{\widetilde{M}P} = \frac{\mathrm{CV}_P}{\mathrm{CV}_{\widetilde{M}}} \frac{1}{2} \left[1 + \frac{1}{\mathrm{CV}_P^2} \left(\frac{1}{2} \frac{\langle P^2 \rangle_{\mathcal{D}}}{\langle P \rangle^2} - 1 - \frac{1}{\langle P \rangle} - \frac{1}{2} \frac{\langle P \rangle_{\mathcal{D}}}{\langle P \rangle^2} \right) \right] \equiv \frac{CV_M}{CV_{\widetilde{M}}} \mathcal{R}. \tag{A.28}$$

The function $DNA(t)$ can be obtained from the Cooper-Helmstetter model. For *not* extremely slow-growing cells $DNA \sim V$, such that $\widetilde{M} \sim M$, hence $\rho_{MP} \simeq \mathcal{R}$.

A.3 Effects of measurement noise on mRNA–protein correlation

We consider multiplicative measurement noise affecting both mRNA and protein abundance, such that the measured mRNA and protein copy numbers are given by $\hat{M} = M(1 + \eta_M)$ and $\hat{P} = P(1 + \eta_P)$, respectively. Here, η_M and η_P are independent Gaussian random variables with zero mean and variances $\sigma^2_{\eta_M}$ and $\sigma^2_{\eta_P}$. The observed mRNA–protein correlation is then

$$\rho_{\hat{M}\hat{P}} = \rho_{MP} \frac{\mathrm{CV}_M \mathrm{CV}_P}{\mathrm{CV}_{\hat{M}} \mathrm{CV}_{\hat{P}}}. \tag{A.29}$$

Substituting ρ_{MP} from Eq. (A.26) yields

$$\rho_{\hat{M}\hat{P}} = \frac{\mathrm{CV}_P^2}{\mathrm{CV}_{\hat{M}} \mathrm{CV}_{\hat{P}}} \frac{1}{2} \left[1 + \frac{1}{\mathrm{CV}_P^2} \left(\frac{1}{2} \frac{\langle P^2 \rangle_{\mathcal{D}}}{\langle P \rangle^2} - 1 - \frac{1}{\langle P \rangle} - \frac{1}{2} \frac{\langle P \rangle_{\mathcal{D}}}{\langle P \rangle^2} \right) \right]. \tag{A.30}$$

Using the relations for the second moments and coefficients of variation,

$$\langle M^2 \rangle = \frac{\langle \hat{M}^2 \rangle}{1 + \sigma^2_{\eta_M}}, \qquad \langle P^2 \rangle = \frac{\langle \hat{P}^2 \rangle}{1 + \sigma^2_{\eta_P}}, \tag{A.31}$$

$$\mathrm{CV}_M = \sqrt{\frac{\mathrm{CV}_{\hat{M}}^2 - \sigma^2_{\eta_M}}{1 + \sigma^2_{\eta_M}}}, \qquad \mathrm{CV}_P = \sqrt{\frac{\mathrm{CV}_{\hat{P}}^2 - \sigma^2_{\eta_P}}{1 + \sigma^2_{\eta_P}}}, \tag{A.32}$$

we obtain

$$\rho_{\hat{M}\hat{P}} = \frac{1}{2\,\mathrm{CV}_{\hat{M}} \mathrm{CV}_{\hat{P}}} \left[\frac{\mathrm{CV}_{\hat{P}}^2 - \sigma^2_{\eta_P}}{1 + \sigma^2_{\eta_P}} + \frac{1}{2(1 + \sigma^2_{\eta_P})} \frac{\langle \hat{P}^2 \rangle_{\mathcal{D}}}{\langle \hat{P} \rangle^2} - 1 - \frac{1}{\langle \hat{P} \rangle} - \frac{1}{2} \frac{\langle \hat{P} \rangle_{\mathcal{D}}}{\langle \hat{P} \rangle^2} \right] \equiv \hat{\mathcal{R}}, \tag{A.33}$$

where we assume that the protein abundance measured at division is affected by multiplicative noise with the same variance $\sigma^2_{\eta_P}$. Notably, the invariant Eq. (A.33) reduces to Eq. (A.26) when protein measurement noise $\sigma_{\eta_P} = 0$, indicating that only protein measurement noise modifies the invariant relation. In other words, although measurement noise in mRNA abundance can influence the observed mRNA–protein correlation, such noise does not affect the invariant relation.

bioRxiv preprint doi: https://doi.org/10.64898/2026.05.04.722628; this version posted May 6, 2026. The copyright holder for this preprint (which was not certified by peer review) is the author/funder, who has granted bioRxiv a license to display the preprint in perpetuity. It is made available under aCC-BY 4.0 International license.

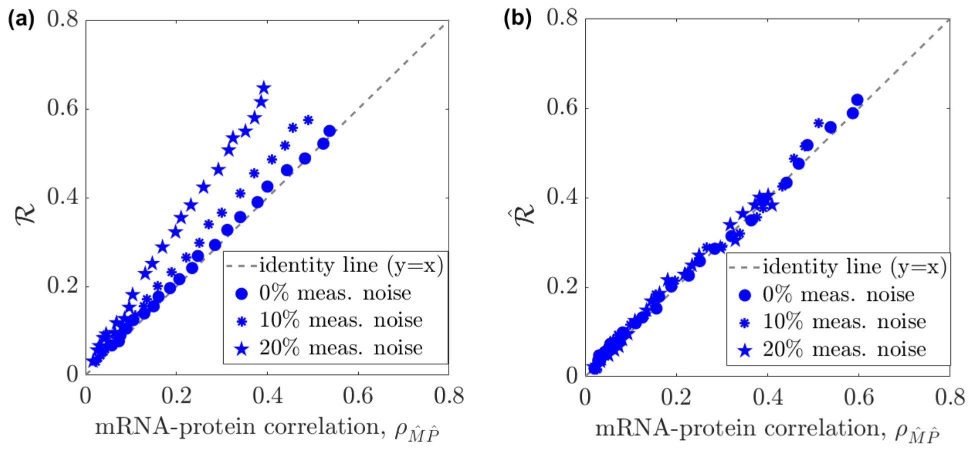

Figure A.2: **Measurement noise attenuates the observed mRNA–protein correlation.** **(a)** In the presence of multiplicative measurement noise in protein copy numbers, the observed correlation $\rho_{\hat{M}\hat{P}}$ is reduced relative to the noiseless prediction of Eq. (A.26), resulting in a systematic deviation from the identity line. **(b)** The correlation in the presence of measurement noise is predicted by Eq. (A.33) and is in good agreement with simulation. Symbols denote different levels of measurement noise, and the dashed line indicates the identity line. The symbols are obtained from stochastic simulation [28] over 10^3 cells.

A.4 Calibration of protein copy number

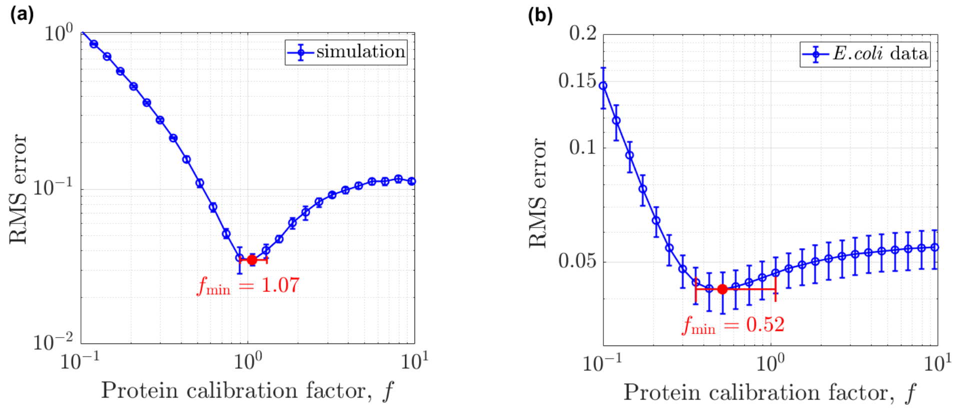

Figure A.3: **Inferring the protein copy-number calibration factor from Eq. (5).** We consider that the true protein copy number P is related to the measured, uncalibrated signal $\hat{P}$ by $P = f\hat{P}$, where f is a constant calibration factor. For each calibration factor f, we compute the root-mean-square (RMS) error for **(a)** simulated data and **(b)** *E. coli* data [11]. The RMS error is defined as $\sqrt{\langle d_i^2 \rangle}$, where $d_i = \mathcal{R} - \rho_{MP}$ is the residual for data point i. ρ_{MP} is the measured mRNA–protein correlation, $\mathcal{R}$ is the corresponding prediction from Eq. (5). The calibration factor is then inferred as the value $f_{\min}$ that minimizes the RMS error. **(a)** For simulated data, $f_{\min} = 1.07$ with a narrow 95% confidence interval of 0.92–1.30. **(b)** For *E. coli* data, $f_{\min} = 0.52$, with a substantially broader 95% confidence interval of 0.36–1.07. We performed bootstrap resampling of the individual sample points, and error bars are 95% bootstrap confidence interval.

bioRxiv preprint doi: https://doi.org/10.64898/2026.05.04.722628; this version posted May 6, 2026. The copyright holder for this preprint (which was not certified by peer review) is the author/funder, who has granted bioRxiv a license to display the preprint in perpetuity. It is made available under aCC-BY 4.0 International license.

B Estimation of $\langle P \rangle_{\mathcal{D}}$ and $\langle P^2 \rangle_{\mathcal{D}}$

To validate the invariant relations in Eq. (5), we need to estimate ρ_{MP} and $\mathcal{R}$ under different conditions. While ρ_{MP} can be obtained directly, estimating $\mathcal{R}$ requires an accurate estimate of the first and second moments of protein number at division, respectively, denoted as $\langle P \rangle_{\mathcal{D}}$ and $\langle P^2 \rangle_{\mathcal{D}}$. However, directly measuring $\langle P \rangle_{\mathcal{D}}$ and$\langle P^2 \rangle_{\mathcal{D}}$ is challenging from population-snapshot data. We therefore infer the protein-number statistics at division as follows.

We define $\langle P^n \rangle_{\mathcal{D}}$ as the n-th moment of the protein number at division:

$$\langle P^n \rangle_{\mathcal{D}} = \frac{\sum_i P_i^n \Pr(\mathcal{D} \mid V_i)}{\sum_i \Pr(\mathcal{D} \mid V_i)}, \tag{B.1}$$

where $\Pr(\mathcal{D} \mid V_i)$ is the probability that a cell with volume V_i divides. Using Bayes' theorem,

$$\Pr(\mathcal{D} \mid V_i) = \frac{\Pr(V_i \mid \mathcal{D}) \Pr(\mathcal{D})}{\Pr(V_i)}, \tag{B.2}$$

which yields

$$\langle P^n \rangle_{\mathcal{D}} = \frac{\sum_i P_i^n \Pr(V_i \mid \mathcal{D}) \frac{\Pr(\mathcal{D})}{\Pr(V_i)}}{\sum_i \Pr(V_i \mid \mathcal{D}) \frac{\Pr(\mathcal{D})}{\Pr(V_i)}} = \frac{\sum_i P_i^n \Pr(V_i \mid \mathcal{D}) / \Pr(V_i)}{\sum_i \Pr(V_i \mid \mathcal{D}) / \Pr(V_i)}. \tag{B.3}$$

Unless stated otherwise, all probabilities in this section refer to population snapshots. The distribution of division volumes in the population, $\Pr(V \mid \mathcal{D})$, is given by

$$\Pr(V \mid \mathcal{D}) = \frac{e^{\mu - \frac{\sigma^2}{2}}}{V^2 \sqrt{2\pi}\,\sigma} \exp\left[-\frac{(\ln V - \mu)^2}{2\sigma^2} \right], \tag{B.4}$$

which follows from the relation

$$\Pr(V \mid \mathcal{D}) = \Pr^{\mathrm{lin}}(V \mid \mathcal{D}) \frac{1}{V \langle 1/V \rangle_{\mathcal{D}}}, \tag{B.5}$$

where $\Pr^{\mathrm{lin}}(V \mid \mathcal{D})$ is the lognormal distribution of division volume along a lineage [44]. Eq. (B.5) gives the relation between the division-volume distributions in the population and lineage ensembles, as reported in Refs. [19, 45].

For given parameters μ and σ, the snapshot distribution of cell volumes, $\Pr(V)$, is obtained from [19, 45]

$$\Pr(V) = \frac{1}{V^2} \int_V^{2V} V' \Pr(V' \mid \mathcal{D})\, dV'. \tag{B.6}$$

Note that in [19, 45] the distribution of division volumes is defined along lineages, whereas here we consistently express everything in terms of population-level probabilities. We estimate μ and σ by maximizing the log-likelihood of the observed snapshot volumes $\{V_i\}$,

$$LL = \sum_i \ln \Pr(V_i). \tag{B.7}$$

With the resulting estimates of μ and σ, we compute $\Pr(V \mid \mathcal{D})$ from Eq. (B.4), $\Pr(V)$ from Eq. (B.6), and subsequently $\langle P \rangle_{\mathcal{D}}$ and $\langle P^2 \rangle_{\mathcal{D}}$ from Eq. (B.3) with $n = 1$ and 2, respectively. We evaluate the performance of this inference procedure using simulated data as ground truth by comparing the inferred $\Pr(V \mid \mathcal{D})$ with the distribution measured directly from the simulations, and find good agreement (Fig. B.1(a)). We then extend the method to *E. coli* data to infer the population division-volume distribution, $\Pr(V \mid \mathcal{D})$, across 11 growth conditions and regulatory architectures, as shown in Fig. B.1(b–l).

bioRxiv preprint doi: https://doi.org/10.64898/2026.05.04.722628; this version posted May 6, 2026. The copyright holder for this preprint (which was not certified by peer review) is the author/funder, who has granted bioRxiv a license to display the preprint in perpetuity. It is made available under aCC-BY 4.0 International license.

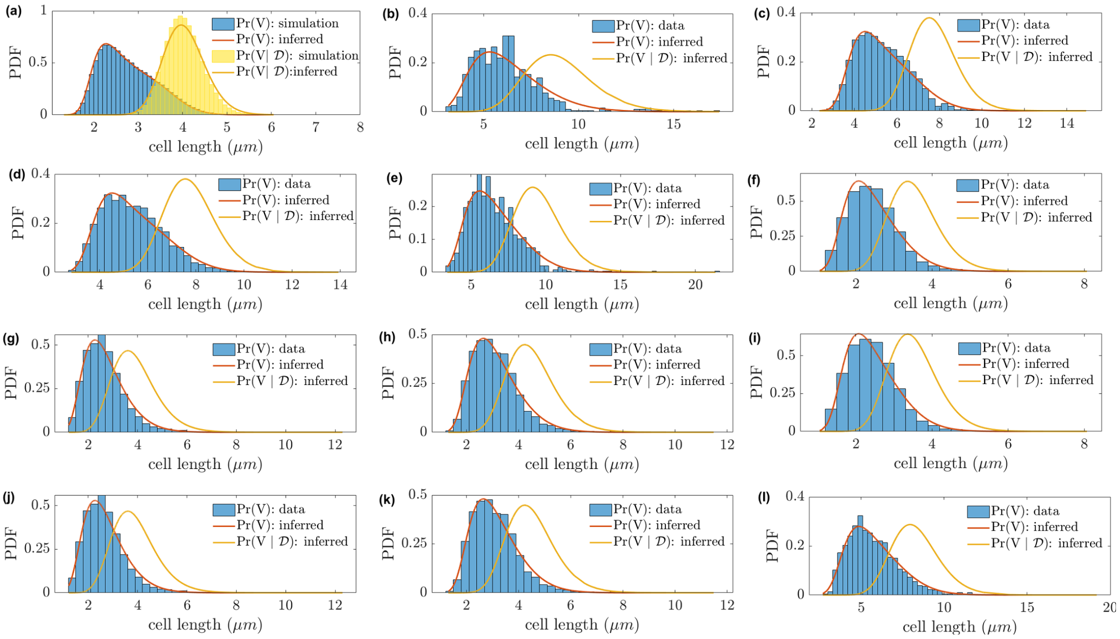

Figure B.1: **Inference of division-size distribution from snapshot data.** **(a)** Validation using simulated data. Blue bars show the snapshot cell-size distribution, $\Pr(V)$, measured directly from the simulations, and the red curve shows the corresponding inferred distribution (Eq. (B.6)). Yellow bars show the division-size distribution in population, $\Pr(V \mid \mathcal{D})$, measured directly from the simulations, and the yellow curve shows the inferred distribution (Eq. (B.4)). Simulations were performed in a growing and dividing cell population, in which each cell elongated, divided into two daughter cells according to an adder size-control mechanism, and partitioned its contents binomially at division, with a constant division time of $T = 50\,\text{min}$. **(b–l)** Application of the same inference procedure to *E. coli* data across 11 growth conditions and regulatory architectures. Blue histograms show the measured snapshot size distributions, the red curves show the inferred $\Pr(V)$, and the yellow curves show the inferred division-size distributions, $\Pr(V \mid \mathcal{D})$.

C Invariant relations for different gene-expression regimes

Protein production is limited by mRNA and/or ribosome levels [7]. Their relative importance varies across conditions, giving rise to three physiological regimes [7, 29]. These gene-expression regimes are schematically illustrated in Fig. C.1(a). We adopt the nomenclature of Ref. [7] and analyze the invariant relation for each regime in turn.

Regime III First, we consider the regime in which transcription is limited only by gene dosage and translation is limited only by mRNA [29]. In this scenario, $\mathit{\Gamma} \propto M$, and the invariant relation in Eq. (A.26) that we analyzed in Sec. A.1 is valid for this regime.

Regimes I and II In Regimes I and II, protein production is limited by both ribosomes, R, and mRNAs. For simplicity, similar to Refs. [7, 29], we assume that all mRNAs have comparable ribosome-binding properties and that ribosomes are allocated in proportion to each mRNA's fraction of the total mRNA pool. Under these assumptions, the total protein production rate is given by

$$\mathit{\Gamma} \propto R \frac{M}{\sum_j M_j}. \tag{C.1}$$

Here, M_j is the mRNA number of gene j, while M is the mRNA number of the gene of interest. Note that the total protein production rate in Eq. (C.1) differs from the simplified assumption

bioRxiv preprint doi: https://doi.org/10.64898/2026.05.04.722628; this version posted May 6, 2026. The copyright holder for this preprint (which was not certified by peer review) is the author/funder, who has granted bioRxiv a license to display the preprint in perpetuity. It is made available under aCC-BY 4.0 International license.

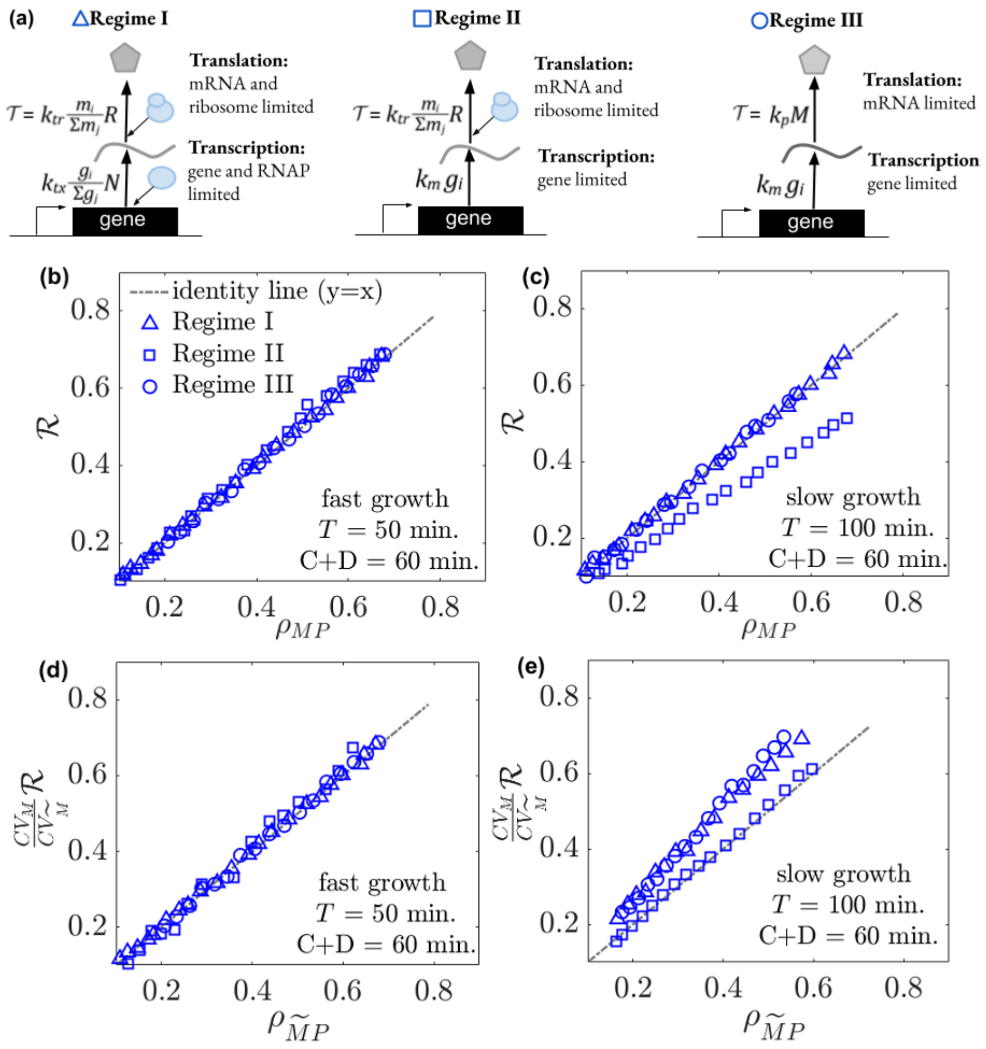

Figure C.1: **Using invariant relations to identify gene-expression regimes in slow growth.** **(a)** Schematic of three different gene expression regimes adapted from [7], where protein and mRNA production rates are limited by different resources: transcription is limited by RNAPs or by the gene dosage, and translation is limited by ribosomes or by the mRNA. The definitions of various model parameters are provided in Appendix Table 1. The invariant Eq. (5) for all three regimes for **(b)** fast and for **(c)** slow growth conditions. In fast-growth ($C + D > T$), all regimes collapse to the same invariant relation, but Regime II deviates from invariant Eq. 5 for slow-growth condition ($C + D < T$). For Regime II, the mRNA–protein correlation measured from stochastic simulation follows the invariant Eq. (A.28) in both **(d)** fast and **(e)** slow growth conditions. However, other regimes deviate from the invariant Eq. (A.28) for slow growth conditions **(e)**. In all plots, symbols are from stochastic simulations [28] over 10^3 cells. In all panels, the dashed line indicates the identity $y = x$ for reference.

$\Gamma \propto M$ used in Sec. A.1, because the prefactor $R/\sum_j M_j$ is not generally constant over the cell cycle. To specify this prefactor, we must characterize the temporal behavior of the total mRNA count, $\sum_j M_j$. This behavior is set by the transcription-limiting factors that govern most genes (not necessarily the gene of interest). We therefore consider two regimes.

Regime I In this regime, the transcription across most genes is limited by the availability of both RNAP number (N) and gene-dosage (g_i) fractions, resulting in $M_i \propto N g_i / \sum_j g_j$. The total mRNA abundance, $\sum_i M_i \propto N \propto V$, therefore scales proportionally with cell volume [46, 47]. Substituting this scaling into Eq. (C.1) yields $\Gamma \propto M$, thereby recovering the same invariant

bioRxiv preprint doi: https://doi.org/10.64898/2026.05.04.722628; this version posted May 6, 2026. The copyright holder for this preprint (which was not certified by peer review) is the author/funder, who has granted bioRxiv a license to display the preprint in perpetuity. It is made available under aCC-BY 4.0 International license.

relation (Eq. A.26) as in regime III and Sec. A.1.

Regime II for fast-growing cells If transcription for most genes is limited primarily by gene dosage, then the total mRNA abundance scales with the total gene copy number, $\sum_j M_j \propto \sum_j g_j$. Under this assumption, Eq. (C.1) is simplified to $\Gamma \propto \frac{RM}{\sum_j g_j}$. The denominator, $\sum_j g_j$, scales with the total cellular DNA content, which increases piecewise linearly during chromosome replication and doubles once per cell cycle [42, 43]. For fast-growing cells, for which the doubling time T is shorter than $C + D$ (where C is the chromosome replication period and D is the time between replication termination and cell division), the piecewise-linear increase in total gene copy number is, in principle, distinguishable from exponential growth given sufficiently precise measurements. Otherwise, when exponential and piecewise linear growths are experimentally indistinguishable, the ratio $R/\sum_j g_j$ is approximately constant throughout the cell cycle. In this case, we again obtain $\Gamma \propto M$, as assumed in Sec. A.1, and recover the same invariant relation, Eq. (A.26), as in Regimes I and III.

Regime II for slow-growing cells In contrast, for slowly growing cells, where $T > C + D$, regime II deviates from Eq. A.26, because for a significant fraction of the cell cycle, total gene number $\sum_j g_j$ does not grow, such that $R/\sum_j g_j$ significantly deviates from a constant. In this case $\Gamma \propto \widetilde{M}$, (where $\widetilde{M} \equiv MV/\sum_j g_j$), the same as in Sec. A.2, and recover, the invariant relation in Eq. (A.28).

Across all regimes considered, we validate the invariant relations using stochastic simulations [28] (see Fig. C.1). Under fast-growth conditions, when $T \lesssim C + D$, all regimes conform to the invariant relation in Eq. (A.26) (Fig. C.1(b)). In contrast, under slow-growth conditions, $T > C + D$, Eq. (A.26) holds for Regimes I and III but not for Regime II (Fig. C.1(c)). In this slow-growth limit, Regime II instead satisfies the invariant relation in Eq. (A.28) (Fig. C.1(e)). These simulations therefore indicate that, at realistic copy numbers and the associated intrinsic noise levels, the regimes remain distinguishable under slow growth (Fig. C.1(c,e)) but are effectively indistinguishable under fast growth (Fig. C.1(b,d)).

bioRxiv preprint doi: https://doi.org/10.64898/2026.05.04.722628; this version posted May 6, 2026. The copyright holder for this preprint (which was not certified by peer review) is the author/funder, who has granted bioRxiv a license to display the preprint in perpetuity. It is made available under aCC-BY 4.0 International license.

D mRNA–protein copy-number versus concentration correlations

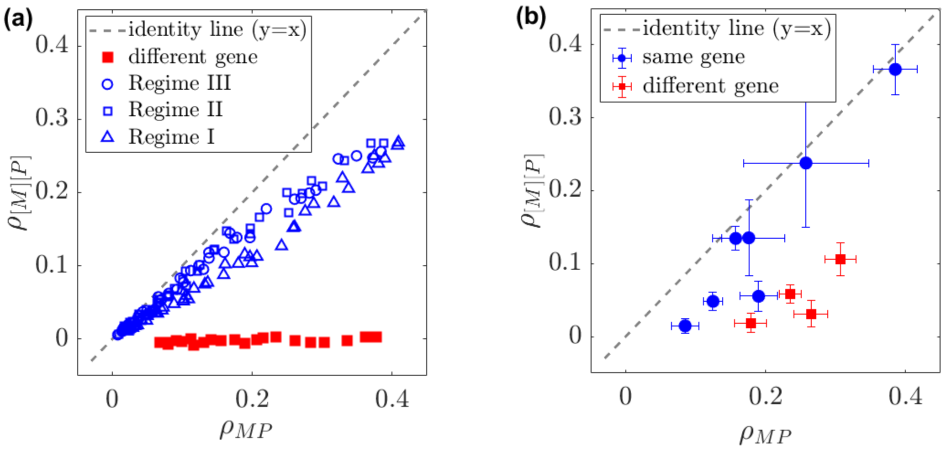

Figure D.1: **Correlation between mRNA and protein *concentrations* versus *copy numbers* in (a) simulations and (b) experimental data. (a)** Simulated mRNA–protein concentrations correlation, $\rho_{[M][P]}$, plotted against the corresponding copy-number correlation, ρ_{MP}, for the same-gene (Regimes I–III; blue symbols) and different-gene pairs (red squares). The dashed line indicates $y = x$. For different-gene pairs, concentration correlations collapse toward zero because the copy-number correlation is mediated solely by cell-volume fluctuations. Same-gene pairs, however, retain a substantial positive concentration correlation, reflecting the direct dependence of protein production on mRNA. **(b)** The same analysis applied to *E. coli* experimental data [11] shows the same trend: concentration correlations remain positive for same-gene pairs, whereas they decrease markedly for different-gene pairs, while remaining above zero, indicating that cell volume is not the sole confounding factor.

E mRNA-protein correlation for unrelated gene pairs

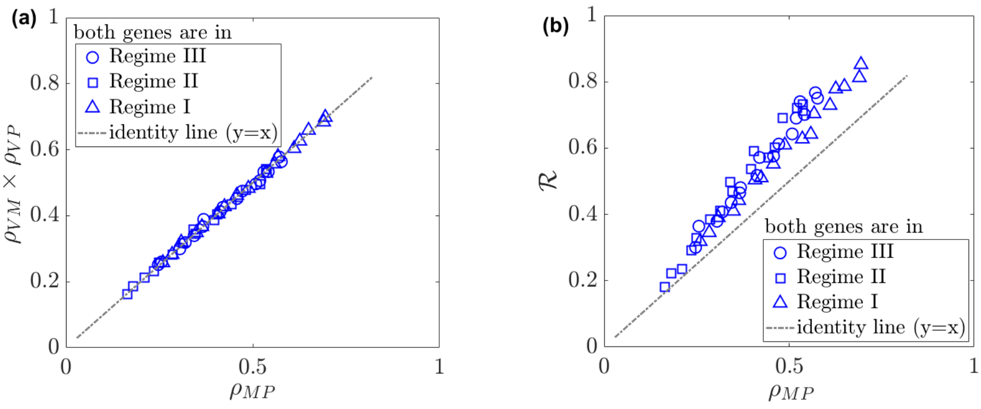

Figure E.1: When mRNA and protein are from two uncorrelated genes, the mRNA–protein correlation reduces to the product of the mRNA–volume correlation (ρ_{VM}) and the protein–volume correlation (ρ_{VP}) because volume is the only confounding factor. This holds for all three gene expression regimes in Sec. C for $T = 50$ min. However, the invariant Eq. (5), $\rho_{MP} = \mathcal{R}$ for the same gene pairs will overestimate the mRNA-protein correlation as expected. In all plots, symbols are from stochastic simulations [28] over 10^3 cells. In both panels, the dashed line indicates the identity $y = x$ for reference.

bioRxiv preprint doi: https://doi.org/10.64898/2026.05.04.722628; this version posted May 6, 2026. The copyright holder for this preprint (which was not certified by peer review) is the author/funder, who has granted bioRxiv a license to display the preprint in perpetuity. It is made available under aCC-BY 4.0 International license.

F Calculation of invariant relations for a lineage of growing and dividing cells

In this scenario, relevant for a mother machine setup [32], a division event results in one daughter cell, the second one is removed from the population. This removal changes the $2\int d\mathbf{y}\mathcal{K}(\mathbf{x}|\mathbf{y})\,\mathcal{D}(\mathbf{y})\mathcal{P}(\mathbf{y},t)$ term in master equation (2) (and Eq. (A.1) in Appendix) to $\int d\mathbf{y}\mathcal{K}(\mathbf{x}|\mathbf{y})\,\mathcal{D}(\mathbf{y})\mathcal{P}(\mathbf{y},t)$ resulting in

$$\frac{d\mathcal{P}(\mathbf{x},t)}{dt} = \sum_k \left[r_k(\mathbf{x}-\mathbf{d}_k)\mathcal{P}(\mathbf{x}-\mathbf{d}_k) - r_k(\mathbf{x})\mathcal{P}(\mathbf{x})\right] + \int d\mathbf{y}\mathcal{K}(\mathbf{x}|\mathbf{y})\,\mathcal{D}(\mathbf{y})\mathcal{P}(\mathbf{y},t) - \mathcal{D}(\mathbf{x})\mathcal{P}(\mathbf{x}). \quad \text{(F.1)}$$

Using the same approach as in Section A, the averages follow

$$\frac{d\langle x_i\rangle}{dt} = \left\langle \sum_k d_{ik} r_k(\mathbf{x}) \right\rangle + \langle x_i \mathcal{D}(2\mathbf{x})\rangle - \langle x_i\mathcal{D}(\mathbf{x})\rangle \quad \text{(F.2)}$$

$$= \left\langle \sum_k d_{ik} r_k(\mathbf{x}) \right\rangle - \frac{1}{2}\langle x_i\mathcal{D}(\mathbf{x})\rangle = \left\langle R_i^+(\mathbf{x})\right\rangle - \left\langle R_i^-(\mathbf{x})\right\rangle - \frac{\lambda_L}{2}\langle x_i\rangle_{\mathcal{D}}, \quad \text{(F.3)}$$

where $\langle x_i\rangle_{\mathcal{D}}$ is the average of x_i at the cell division and λ_L is the division rate averaged along the lineage

$$R_i^+ = \sum_{k:d_{ik}>0} d_{ik} r_k(\mathbf{x}) \text{ and } R_i^- = \sum_{k:d_{ik}<0} |d_{ik}| r_k(\mathbf{x}). \quad \text{(F.4)}$$

At the stationarity we have a bias towards the positive rate to balance the cell divisions:

$$\left\langle R_i^+\right\rangle - \left\langle R_i^-\right\rangle = \frac{\lambda_L}{2}\langle x_i\rangle_{\mathcal{D}}. \quad \text{(F.5)}$$

The second moments follow

$$\frac{d\langle x_i x_j\rangle}{dt} = \sum_k \left[d_{ik}d_{jk}\langle r_k(\mathbf{x})\rangle + d_{jk}\langle x_i r_k(\mathbf{x})\rangle + d_{ik}\langle x_j r_k(\mathbf{x})\rangle\right] - \frac{3}{4}\lambda_L\langle x_i x_j\rangle_{\mathcal{D}} + \frac{\lambda_L}{2}\delta_{ij}\langle x_i\rangle_{\mathcal{D}}, \quad \text{(F.6)}$$

$$= \sum_k d_{ik}d_{jk}\langle r_k(\mathbf{x})\rangle + \left\langle x_i(R_j^+ - R_j^-)\right\rangle + \left\langle x_j(R_i^+ - R_i^-)\right\rangle - \frac{3}{4}\lambda_L\langle x_i x_j\rangle_{\mathcal{D}} + \frac{\lambda_L}{2}\delta_{ij}\langle x_i\rangle_{\mathcal{D}}, \quad \text{(F.7)}$$

$$= B_{ij} + \left\langle (x_i - \langle x_i\rangle)(R_j^+ - R_j^-)\right\rangle + \lambda_L\frac{\langle x_j\rangle_{\mathcal{D}}\langle x_i\rangle}{2} \quad \text{(F.8)}$$

$$+ \left\langle (x_j - \langle x_j\rangle)(R_i^+ - R_i^-)\right\rangle + \lambda_L\frac{\langle x_i\rangle_{\mathcal{D}}\langle x_j\rangle}{2} - \frac{3}{4}\lambda_L\langle x_i x_j\rangle_{\mathcal{D}} + \frac{\lambda_L}{2}\delta_{ij}\langle x_i\rangle_{\mathcal{D}}, \quad \text{(F.9)}$$

$$= B_{ij} + \mathrm{Cov}\left(x_j, R_i^+\right) - \mathrm{Cov}\left(x_j, R_i^-\right) + \mathrm{Cov}\left(x_i, R_j^+\right) - \mathrm{Cov}\left(x_i, R_j^-\right) \quad \text{(F.10)}$$

$$+ \lambda_L\frac{\langle x_j\rangle_{\mathcal{D}}\langle x_i\rangle + \langle x_i\rangle_{\mathcal{D}}\langle x_j\rangle}{2} - \frac{3}{4}\lambda_L\langle x_i x_j\rangle_{\mathcal{D}} + \frac{\lambda_L}{2}\delta_{ij}\langle x_i\rangle_{\mathcal{D}} = 0. \quad \text{(F.11)}$$

For $i = j$ we get

$$\langle R_i^+ + R_i^-\rangle + 2\mathrm{Cov}\left(x_i, R_i^+\right) - 2\mathrm{Cov}\left(x_i, R_i^-\right) + \lambda_L\langle x_i\rangle_{\mathcal{D}}\langle x_i\rangle - \frac{3}{4}\lambda_L\langle x_i^2\rangle_{\mathcal{D}} + \frac{\lambda_L}{2}\langle x_i\rangle_{\mathcal{D}} = 0. \quad \text{(F.12)}$$

As before, for $i = 2$, $x_2 = P$, $R_2^- = \gamma_p P$ and $R_2^+ = \Gamma$ we get

$$\mathrm{Cov}\left(P, \Gamma\right) = \gamma_p\mathrm{Var}(P) - \frac{\lambda_L}{2}\langle P\rangle_{\mathcal{D}}\langle P\rangle + \frac{3}{8}\lambda_L\langle P^2\rangle_{\mathcal{D}} - \frac{\lambda_L}{4}\langle P\rangle_{\mathcal{D}} - \frac{\langle\Gamma\rangle + \gamma_p\langle P\rangle}{2}, \quad \text{(F.13)}$$

and using Eq. (F.5) $\langle\Gamma\rangle = \frac{\lambda_L}{2}\langle P\rangle_{\mathcal{D}} + \gamma_p\langle P\rangle$

$$\rho_{\Gamma P} = \frac{1}{\mathrm{CV}_\Gamma\mathrm{CV}_P}\frac{\gamma_p\langle P\rangle^2\mathrm{CV}_P^2 - \frac{\lambda_L}{2}\langle P\rangle_{\mathcal{D}}\langle P\rangle + \frac{3}{8}\lambda_L\langle P^2\rangle_{\mathcal{D}} - \frac{\lambda_L}{2}\langle P\rangle_{\mathcal{D}} - \gamma_p\langle P\rangle}{\frac{\lambda_L}{2}\langle P\rangle_{\mathcal{D}}\langle P\rangle + \gamma_p\langle P\rangle^2}. \quad \text{(F.14)}$$

bioRxiv preprint doi: https://doi.org/10.64898/2026.05.04.722628; this version posted May 6, 2026. The copyright holder for this preprint (which was not certified by peer review) is the author/funder, who has granted bioRxiv a license to display the preprint in perpetuity. It is made available under aCC-BY 4.0 International license.

For $\gamma_p \ll \lambda_L$ this is simplified to

$$\rho_{\Gamma P} = \frac{1}{\mathrm{CV}_\Gamma \mathrm{CV}_P} \frac{\frac{3}{4}\langle P^2\rangle_{\mathcal{D}} - \langle P\rangle_{\mathcal{D}} - \langle P\rangle_{\mathcal{D}}\langle P\rangle}{\langle P\rangle_{\mathcal{D}}\langle P\rangle} \equiv \mathcal{R}^{MM}. \tag{F.15}$$

As before, for fast-growing cells, the invariant relation for all regimes

$$\rho_{MP} = \mathcal{R}^{MM}, \tag{F.16}$$

(see Fig. F.1(a)), and, otherwise, in regime II, the invariant relation,

$$\rho_{\widetilde{M}P} = \frac{\mathrm{CV}_M}{\mathrm{CV}_{\widetilde{M}}}\mathcal{R}^{MM}, \tag{F.17}$$

see Fig. F.1(b). It is important to note that the mRNA-protein correlation for the lineage in Eq. (F.15) follows a different invariant relation than that of the tree ensemble in Eq. (4) as shown in Fig. F.1(c). This distinction highlights that mRNA–protein correlations depend on the observational ensemble, and that predictions obtained for lineage measurements cannot, in general, be directly identified with those for tree-ensemble statistics.

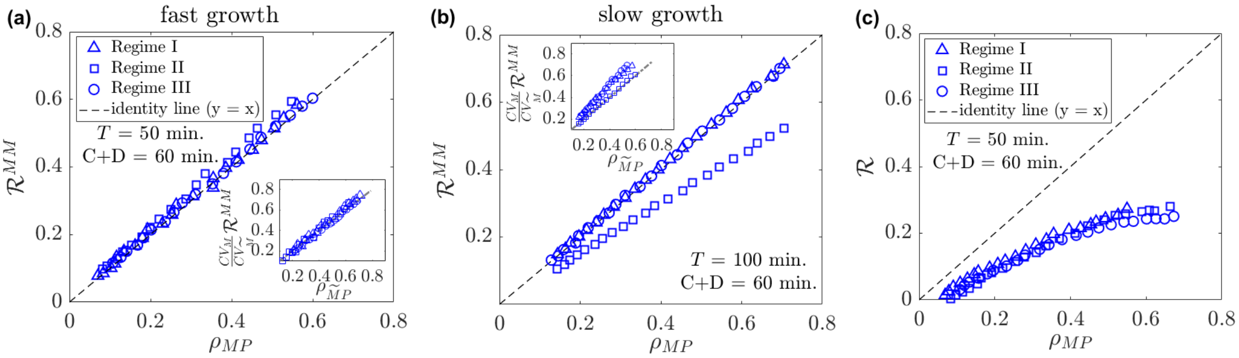

Figure F.1: **Invariant relations for a lineage across three different gene expression regimes.** **(a)** For fast growth conditions, regimes are indistinguishable and follow the invariant relation in Eq. (F.16). Inset: all regimes will also follow the other invariant relation in Eq. (F.17) in the fast growth condition. **(b)** In the slow growth condition, Regime II will differ from invariant relation in Eq. (F.16) and follow the invariant relation in Eq. (F.17) (inset figure). However, Regimes I and III will differ from Eq. (F.17) in slow growth conditions (inset figure). **(c)** mRNA-protein correlation for cells within a lineage differs from the invariant relation for the tree ensemble in Eq. (5), indicating that the sampling method for cells matters for the mRNA-protein correlation. In all plots, symbols are from stochastic simulations [28] over 10^3 cells. In both panels, the dashed line indicates the identity $y = x$ for reference.

bioRxiv preprint doi: https://doi.org/10.64898/2026.05.04.722628; this version posted May 6, 2026. The copyright holder for this preprint (which was not certified by peer review) is the author/funder, who has granted bioRxiv a license to display the preprint in perpetuity. It is made available under aCC-BY 4.0 International license.

Symbol	Description
k_m	Transcription rate
k_{tx}	Transcription rate per RNA polymerase (RNAP)
k_p	Translation rate
k_{tr}	Translation rate per ribosome
γ_m	mRNA degradation rate
γ_p	Protein degradation rate
$\mathit{\Gamma}$	Total protein production rate
$\mathbf{x}$	cell state
$\mathcal{D}(\mathbf{x})$	Division rate
$R^-_{\mathcal{P}}(\mathbf{x})$	Protein degradation flux
$\mathcal{K}(\mathbf{x}\|\mathbf{y})$	Binomial partition kernel, i.e., the conditional probability that a daughter cell is born in state $\mathbf{x}$ given that the mother cell divided in state $\mathbf{y}$
λ	Population growth rate
M	mRNA copy number
P	Protein copy number
R	Ribosome copy number
N	RNA polymerase (RNAP) copy number
g_i	Gene copy number
V	Cell volume
$\langle P\rangle_{\mathcal{D}}$	Mean protein copy number at cell division
T	Cell division time
C period	Time from replication initiation to replication termination
D period	Time from replication termination to cell division
$\langle\cdot\rangle$	Population average
σ^2	Variance
CV	Coefficient of variation (defined as standard deviation divided by mean)
ρ_{MP}	Pearson correlation coefficient between mRNA and protein copy numbers
$\mathcal{P}(\mathbf{x}, t)$	Probability density of the cell state $\mathbf{x}$ at time t

Table 1: Description of symbols used throughout the manuscript.
